# Pan-negative型非小细胞肺癌的研究进展

**DOI:** 10.3779/j.issn.1009-3419.2018.02.07

**Published:** 2018-02-20

**Authors:** 丽 孙, 志成 熊, 琤波 韩

**Affiliations:** 110022 沈阳, 中国医科大学盛京医院肿瘤内科 Department of Oncology, Shengjing Hospital of China Medical University, Shenyang 110022, China

**Keywords:** 肿瘤, 驱动基因, Pan-negative, Lung neoplasms, Driver gene, Pan-negative

## Abstract

近年来随着对肿瘤驱动基因的不断探索和分子检测技术的快速发展，在非小细胞肺癌（non-small cell lung cancer, NSCLC）领域，一系列的驱动基因如*EGFR*、*KRAS*、*BRAF*、*PIK3CA*、*ALK*和*ROS-1*等相继被发现，并逐渐研发出相应的针对特定驱动基因变异的靶向治疗药物，使NSCLC患者的生存得到极大改善。尽管如此，仍有部分NSCLC患者未能检测到任何已知驱动基因变异，称之为pan-negative型NSCLC。本文就pan-negative型NSCLC的概念、临床病理和流行病学特点以及治疗预后等作一综述。

## Abstract (en)

In recent years, series of driver genes, such as *EGFR, KRAS/NRAS, BRAF, PIK3CA, ALK* and *ROS1* and so on, have been found in non-small cell lung cancer (NSCLC) one after another with the development of molecular detecting technology. Targeted drugs bring benefits for these NSCLC patients with driver gene variations. However, some NSCLC did not have any known driver gene variations; we called it pan-negative lung cancer. In this paper, we summarize the concept, clinical pathological characteristics, the epidemiological characteristics, treatment and prognosis of pan-negative NSCLC.

非小细胞肺癌（non-small cell lung cancer, NSCLC）约占肺癌患者的85%，是世界范围内癌症相关死亡的主要原因^[1]^。随着分子检测技术不断提高，越来越多驱动基因被发现。研究^[2]^表明，70%以上的NSCLC具有明确的驱动基因，包括*EGFR*、*ALK*、*ROS-1*、*BRAF*、*KRAS*、*MET*、*PIK3CA*、*RET*、*HER-2*和*MEK1/2*基因等。针对一些驱动基因变异的靶向药物相继上市，如治疗*EGFR*基因敏感突变的厄洛替尼、吉非替尼和阿法替尼，以及EGFR T790M耐药突变的奥希替尼；针对*ALK*和*ROS-1*基因重排的克唑替尼、色瑞替尼（ceritinib）和阿雷替尼（alectinib）；以及针对*BRAF* V600E突变的达拉菲尼（dabrafenib）和曲美替尼（trametinib）联合疗法等，具体参见表 1。肺鳞癌中约有20%携带有*FGFR1*扩增，约2%发生*DDR2*突变。*DDR2*突变的肺鳞癌对达沙替尼（dasatinib）具有一定敏感性^[3]^。相比传统化疗，靶向药物应答率更高，患者无进展生存时间（progression-free survival, PFS）更长。目前针对其他驱动基因变异的靶向治疗药物也在临床实验中不断探索^[4]^。尽管如此，目前仍有部分NSCLC患者即便采用新一代测序技术（next generation sequencing, NGS）对其全基因组分析检测，仍然检测不到任何驱动基因变异，称之为pan-negative型NSCLC。本文就pan-negative型NSCLC患者的概念、临床病理和流行病学特点及治疗预后等作一综述。

**1:** NSCLC常见驱动基因、变异形式以及目前国内外批准上市的药物 The common driver genes of NSCLC, variation types and drugs currently approved for sale

Driver genes	Variation types	Variationrates		Target inhibitors	IC_50_ value （nmol/L）	Approved by CFDA	Approved by FDA
		Asians	Caucasians				
*EGFR*	Mutation	30%-40%^[3, 4]^	10%-15%^[3, 4]^	Gefitinib	2-37	Y	Y
				Erlotinib	2	Y	Y
				Icotinib	5	Y	N
				Afatinib	0.4^d^	Y	Y
				Osimertinib^a^	1	Y	Y
				Avitinib^b^	0.18	N	N
				Rociletinib^a^	62-187	N	N
*ALK*	Rearrangement	≤4%^[3]^		Crizotinib	24	Y	Y
				Ceritinib	0.2	N	Y
				Alectinib	1.9	N	Y
				Brigatinib	0.6	N	Y
				Ioratinib	0.7	N	N
*ROS-1*	Rearrangement	1%-3%^[3]^		Crizotinib	-	Y	Y
				loratinib	< 0.02	N	N^e^
				Ceritinib	-	N	N
*MET*	Amplification	1%-7% primary or 20%		Crizotinib	11	N	N^e^
		acquired^[3]^		Capmatinib	0.13±0.05	N	N
	Exon 14 skipping Mutation	3%^[3]^		Tivantinib	0.355 *μ*mol/L (Ki)	N	N
*HER-2*	Amplification	2%^[3, 4]^		Afatinib	14	N	N
				Trastuzumab	NR	N	N^e^
	Mutation	1.6%-4%^[3]^		T-DM1^c^	NR	N	N
*BRAF*	Mutation (V600E)	1.6%^[5]^	3.5%^[5]^	Vemurafenib	31	N	N
				Dabrafenib	0.6	N	Y^f^
*RET*	Rearrangement	0.7%-2%^[7, 8]^		Cabozantinib	4	N	N
				Vandetanib	40	N	N
*MEK1/2*	Mutation	1%^[6]^		Trametinib	2	N	N
				Selumetinib	14	N	N
Y: yes; N: no; NR: not reported; a: *EGFR* sensitive mutation and T790M resistant mutation; b: *EGFR* L858R/T790M double mutations; c: T-DM1, ado-trastuzumab emtansine; d: L858R; e: recommended by NCCN guideline; f: dabrafenib combined with trametinib has been approved by FDA to treat NSCLC with *BRAF* V600E mutation; NSCLC: non-small cell lung cancer; NCCN: National Comprehensive Cancer Network; FDA: Food and Drug Administration; EGFR: epidermal growth factor receptor.							

## 1 Pan-negative概念

研究表明，NSCLC不同组织类型的驱动基因分类差别较大。肺腺癌的主要驱动基因变异有*EGFR*、*HER2*、*KRAS/NRAS*、*BRAF*、*PI3KCA*和*HER-2*等基因突变，*ALK*、*ROS1*和*RET*基因重排，以及*MET*基因扩增等^[2]^；肺鳞癌的主要驱动基因包括*FGFR1*、*DDR2*、*SOX*、*MDM2*、*PDGFRA*和*P53*等基因突变、PTEN缺失等^[5-9]^。尽管目前已经发现上述多种肺癌驱动基因变异，但仍有部分NSCLC患者未检测到任何驱动基因变异。这种癌组织或细胞经全基因组序列分析或联合蛋白水平检测未发现任何目前已知的驱动基因变异的肺癌类型被称之为pan-negative型NSCLC。

“Pan-negative”一词最早见于黑色素瘤和NSCLC中。Hutchinson等曾描述大约35%的黑色素瘤被认为是广泛阴性的（Pan-negative），即缺乏已知驱动基因*BRAF*、*NRAS*、*KIT*、*GNAQ*、*GAN11*中的任何一种变异^[10]^。2010年上海复旦大学肿瘤医院对52例不吸烟肺腺癌术后标本采用序列分析，检测其*EGFR*、*HER2*、*KRAS*、*BRAF*、*TP53*、*LKB1*、*PIK3CA*突变以及*ALK*融合。结果发现有接近10%的标本缺乏驱动基因变异。该团队将这类经测序方法检测不到已知驱动基因变异的肺癌类型称之为“pan-negative”肺癌^[11]^。2012年Li等^[12]^利用全外显子序列（Affymetrix Exon 1.0）分析方法重新检测上述“pan-negative”肺癌标本并对可能存在的融合激酶进行热谱分析，结果发现在这些“pan-negative”肺癌标本中发现其中1例存在*RET*融合，1例存在*ROS1*融合，因此这52例肺癌标本的驱动基因阴性比例由9.6%变成5.8%。因此pan-negative状态并非固定不变，尤其是在疾病进展或经治疗后，应考虑到其空间异质性和时间异质性，即个体同一疾病的任一空间病灶或任一时间点检测出驱动基因变异均可以排除此时pan-negative状态。正如目前所报道的pan-negative的病例大多为肺腺癌中未检测到某些特定驱动基因变异，每篇报道所检测的驱动基因数目也不尽相同，从几个到十几个不等。因而，这种在癌组织/细胞中未检测出任何预先规定的驱动基因变异类型，我们称之为狭义的pan-negative型NSCLC（需要指明特定组织和标本类型）。

## 2 Pan-negative型NSCLC流行病学特点

由于各项研究的病例样本、人群分布、检测手段，以及检测驱动基因变异数目的差异，造成pan-negative型NSCLC的检出率并不一致。对pan-negative型肺腺癌和肺鳞癌的国内外流行病学研究情况分述如下。

2013年美国临床肿瘤学会（American Society of Clinical Oncology, ASCO）会议上，美国肺癌突变联盟（Lung Cancer Mutation Consortium, LCMC）报道了一项肺腺癌基因变异谱的研究，对1, 007例Ⅲb期或Ⅳ期肺腺癌患者进行了10种已知癌症驱动基因的检测（包括*KRAS*、*EGFR*、*BRAF*、*PIK3CA*、*AKT1*、*MEK1*、*NRAS*突变，*ALK*重排和*HER2*、*MET*扩增）。结果发现，在773例肺腺癌中，高达36%的样本未检测出上述已知的驱动基因变异^[13]^。另一项是Philip团队针对美国非洲裔NSCLC患者驱动基因谱的研究，对10个已知驱动基因（包括*EGFR*、*KRAS*、*BRAF*、*NRAS*、*AKT1*、*PIK3CA*、*PTEN*、*ERBB2*和*MEK1*基因突变，*ALK*重排）进行了检测，结果发现非鳞型NSCLC中pan-negative的比例为22%（35/161）^[14]^。此外，法国生物标志物研究（BM）团队开展了迄今为止样本量最大的驱动基因变异谱研究，对10, 000例生物标记物数据进行了6个已知驱动基因变异（包括*EGFR*、*KRAS*、*BRAF*、*HER2*和*PIK3CA*基因突变，*ALK*基因重排）的检测，结果发现高达55%的样本属于基因变异未知人群^[15]^。Custom研究是首个评估生物标志物配对治疗疗效的前瞻性临床研究，检测入组的482例NSCLC患者的已知驱动基因（包括*EGFR*、*KRAS*、*PIK3CA*、*BRAF*、*HRAS1*、*ERBB2*、*AKT1*、*NRAS*基因突变，*HER2*扩增，*PTEN*缺失，*ALK*重排），发现pan-negative型NSCLC比例达36%^[16]^。

国内研究方面，2012年吴一龙团队采用聚合酶链反应（polymerase chain reaction, PCR）和直接测序等方法对524例NSCLC的10个驱动基因变异（包括*EGFR*、*KRAS*、*BRAF*、*STK11*、*PIK3CA*、*DDR2*、*FGFR2*基因突变，*PTEN*缺失、*MET*扩增、*ALK*重排）进行了检测，结果显示只有10.9%的患者未检测出明确的驱动基因变异^[17]^。另一项中国的研究来自上海复旦大学癌症中心，研究者同样采用PCR和直接测序等方法检测了近两千例NSCLC患者癌组织的7个驱动基因变异（包括*EGFR*、*KRAS*、*HER2*、*BRAF*基因突变，*ALK*、*ROS1*、*RET*融合）。在1, 356例肺腺癌当中，pan-negative的比例占17.3%^[18]^。而对于不吸烟的肺腺癌患者，Li等^[19]^报道了只有12%（24/202）的东亚裔不吸烟肺腺癌患者未能检测出任何驱动基因变异。该研究还发现存在未知基因突变的患者与具有已知突变的患者相比年龄偏小（52.3岁*vs* 57.9岁，*P*=0.013）。

从上述国内外的研究显示，东西方pan-negative型肺腺癌发生率有所差异，东亚裔pan-negative型NSCLC的发生率（10%-20%）似乎低于高加索人群（20%-50%）。关于pan-negative型肺腺癌的临床病理特征目前尚缺乏准确的大宗数据报道，有待于进一步研究证实。现阶段的普遍认识是，pan-negative型肺腺癌是一个独特的分子亚型，可能具有不同于其他肺腺癌的临床病理学特征。

关于肺鳞癌驱动基因的研究也从未停止。最近一项日本的前瞻性肺鳞癌驱动基因变异谱研究，检测了129例肺鳞癌和腺鳞癌患者的12项基因变异（包括*EGFR*、*KRAS*、*BRAF*、*PIK3CA*、*NRAS*、*MEK1*、*AKT1*、*HER2*和*DDR2*基因突变，*ALK*重排，*PTEN*缺失，*ROS1*融合），发现大约50%样本未检测出上述驱动基因变异。不同的标本类型检测结果不同，石蜡包埋的标本基因变异检出率低于术中快速冰冻标本（29% *vs* 50%）。该研究中还发现在鳞癌和腺鳞癌人群中pan-negative发生率与患者年龄和吸烟状态有关，年龄大于70岁（48% *vs* 35%）和吸烟者（78% *vs* 10%）pan-negative发生率高^[20]^。美国国家癌症研究所（National Cancer Institute, NCI）与美国西南肿瘤组（Southwest Oncology Group, SWOG）牵头推出了生物标志物驱动的肺鳞癌新药的前瞻性研究（lung-MAP, SWOG S1400），基于晚期肺鳞癌的5个基因检测结果将患者分配到5个子研究中。在这些子研究中，患者或接受标准治疗或接受针对相应基因变异的靶向治疗。该研究有望为有关肺鳞癌的驱动基因及靶向治疗带来新的答案^[21]^。

## 3 Pan-negative型NSCLC的治疗及预后

LCMC的一项针对肺腺癌驱动基因变异谱的研究显示，筛查到驱动基因变异且接受相应靶向治疗者、筛查到驱动基因未接受靶向治疗者，以及无驱动基因无靶向治疗者的中位OS分别为3.5年、2.4年和2.1年，提示pan-negative型NSCLC预后生存并不比含有驱动基因的患者预后差^[13]^。表 2所列为Pan-negative型NSCLC目前国内外各指南推荐的治疗模式选择。

**2:** 目前各指南推荐的Pan-negative型晚期NSCLC治疗选择 Current guidelines recommended for the treatment of advanced Pan-negative NSCLC

Guideline	Pathology	First line	Maintenance	Second line	Third line
NCCN 2018 v2^[55]^	Squamous carcinoma	PD-L1≥50%: PS 0-2: Pembrolizumab. If progress, treatment according to NSCLC of PD-L1 < 50%; PS 3-4: Best supportive care	-	PS 0-1: Platinum-based two drugs chemotherapy; PS 2: Two drugs combined or single drug chemotherapy PS 3-4: Best supportive care	PS 0-2: Docetaxel+ramucirumab/gemcitabine; PS 3-4: Best supportive care
		PD-L1 < 50%: PS 0-1: Platinum-based chemotherapy；PS 2: Two drugs combined or single drug chemotherapy PS 3-4: Best supportive care	Gemcitabine or docetaxel	PS 0-2: Systemic immune checkpointinhibitors^A^; docetaxel+ramucirumab; gemcitabine; PS 3-4: Best supportive care	PS 0-2: Therapyneverused before PS 3-4: Best supportive care
	Non-squamous carcinoma	PD-L1≥50%: Same as above	-	PS 0-1: Platinum-based two drugs chemotherapy+bevacizumab; Pemetrexed+carboplatin+pembrolizumab PS 2: Platinum-based two drugs or single drug chemotherapy	PS 0-2: Docetaxel+ramucirumab/gemcitabine; pemetrexed PS 3-4: Best supportive care
		PD-L1 < 50%: PS 0-1: Platinum-based two drugs chemotherapy+bevacizumab; Pemetrexed+carboplatin+ pembrolizumab PS 2: Platinum-based two drugs or single drug chemotherapy PS 3-4: Best supportive care	Bevacizumab; pemetrexed; pemetrexed + bevacizumab; gemcitabine	PS 0-2: Systemic immune checkpointinhibitors^A^; docetaxel+ ramucirumab; gemcitabine; pemetrexed PS 3-4: Best supportive care	PS 0-2: Therapy neverused before PS 3-4: Best supportive care
ESMO Guideline 2016^[56]^	Squamous carcinoma	Age < 70 and PS 0-1: Platinum-based two drugs (4-6 cycles) orcisplatin+gemcitabine+necitumumab (EGFR IHC+)	-	PS 0-2: Systemic immune checkpoint inhibitors^B^/ docetaxel + ramucirumab; erlotinib/afatinib	-
		Age≥70 and PS 2 or age < 70 and PS 0-2: Carboplatin-based two drugs or single drug chemotherapy^C^		PS 3-4: Best supportive care；	
		PS 3-4: Best supportive care			
	Non-squamous carcinoma	Age < 70 and PS 1: Platinum-based two drugs (4-6 cycles) orcisplatin+albumin taxol+ bevacizumab	Pemetrexed+ bevacizumab	PS 0-2: Systemic immune checkpoint inhibitors^B^/pemetrexed /docetaxel+ramucirumab/nintedanib/ erlotinib	-
		Age≥70 and PS 2 or age < 70 and PS 0-2: Carboplatin-based two drugs or single drug chemotherapy^C^			
		PS 3-4: Best supportive care		PS 3-4: Best supportive care	
CSCO Guideline 2017 v1^[57]^	Squamous carcinoma	PS 0-1: Platinum-based two drugs/ gemcitabine+docetaxel/vinorelbine	-	PS 0-2: Docetaxel/vinorelbine/gemcitabine/ afatinib	-
		PS 2: Single drug chemotherapy^D^		PS 3-4: Clinical trial or best supportive care	
		PS 3-4: Clinical trial or best supportive care			
	Non-squamous carcinoma	PS 0-1: Platinum-based two drugs chemotherapy+bevacizumab	Pemetrexed；bevacizumab	PS 0-2: Docetaxel/pemetrexed	Clinical trial or best supportive care
		PS 2: Single drug chemotherapy^D^; pemetrexed+ carboplatin ortaxol+carboplatin (every week)			
		PS 3-4: Clinical trial or best supportive care		PS 3-4: Best supportive care	
A: nivolumab, pembrolizumab（PD-L1≥1%）and atezolizumab; B: nivolumab, pembrolizumab (PD-L1≥1%); C: vinorelbine, gemcitabineand docetaxel; D: vinorelbine, gemcitabine, docetaxel, and taxol.					

### 3.1 一线治疗

在一线治疗中，KEYNOTE-024研究证实在未经治疗且PD-L1表达强阳性（≥50%）的NSCLC患者中，无论是ORR、PFS还是OS，帕姆单抗（pembrolizumab）均显著优于传统的一线含铂两药化疗方案，且安全性更好^[22]^。基于该研究，NCCN指南目前推荐帕姆单抗为PD-L1表达强阳性NSCLC一线首选治疗。

针对PD-L1 < 50且无靶向驱动基因变异的NSCLC，以铂类为基础的两药联合化疗通常是目前一线治疗的基石。ECOG4599和BEYOND两项Ⅲ期随机对照研究以及最近的一项荟萃分析表明，以铂类为基础的化疗联合贝伐单抗一线治疗晚期非鳞型NSCLC对比单纯化疗能够显著延长患者的OS和PFS。因此化疗联合贝伐单抗也成为目前无驱动基因变异NSCLC的一线治疗重要选择^[23-25]^。最近的一项KEYNOTE-021G队列研究显示，对于任何程度PD-L1表达的晚期不携带*EGFR*敏感突变或*ALK*基因重排的非鳞型NSCLC，在一线卡铂联合培美曲塞标准化疗基础上加入Pembrolizumab可显著提高患者的ORR、延长PFS。KEYNOTE-021G研究为免疫治疗联合化疗治疗非鳞型NSCLC提供了初步的证据^[26]^，但仍需Ⅲ期随机对照研究加以验证。IMpower150研究（NCT02366143）评估了免疫治疗联合化疗（紫杉醇+卡铂）联合或不联合抗血管药物（贝伐珠单抗）一线治疗Ⅳ期无驱动基因变异的非鳞NSCLC的疗效和安全性。该研究纳入1, 202例患者随机分为阿特珠单抗+化疗方案（A组）与阿特珠单抗+化疗+贝伐珠单抗方案（B组）及化疗+贝伐珠单抗方案（C组）。各组化疗4-6周期，A组用单药阿特珠单抗维持治疗，B组用贝伐单抗联合阿特珠单抗维持治疗，C组用贝伐珠单抗维持治疗。中期结果分析了B组和C组数据，结果发现三联疗法中位PFS、总体缓解率ORR较贝伐珠单抗联合化疗组优势显著（PFS：8.3个月*vs* 6.8个月，HR：0.62，*P* < 0.001；ORR：64% *vs* 48%），OS数据有待研究继续进行^[27]^。该研究为无驱动基因变异的非鳞型NSCLC的一线治疗打开新的篇章。更多PD-1/PD-L1抑制剂与抗血管靶向治疗、CTLA-4抑制剂或放疗的联合治疗也正在积极探索中。

业已证实，EGFR野生型NSCLC大多不能从EGFR-TKI治疗中获益，但EGFR信号通路仍然在EGFR野生型肺癌细胞的增殖、侵袭和转移中扮演重要角色。EGFR单抗，如西妥昔单抗和necitumumab等，可与肿瘤细胞表面EGFR有效结合，抑制EGFR下游信号通路。FLEX研究首次证实，对经免疫组化证实EGFR强阳性的晚期NSCLC患者采用西妥昔单抗联合化疗（顺铂和长春瑞滨）相比单纯化疗，OS得以改善（12个月*vs* 9.6个月，*P*=0.011）^[28]^。2017年*Lancet Oncology*发布的Ⅲ期临床研究SWOG S0819中，在标准化疗基础上联合西妥昔单抗并不能延长EGFR FISH阳性晚期NSCLC患者的PFS和总体人群的OS^[29]^。因此，西妥昔单抗在NSCLC治疗中的价值及获益人群还有待进一步探索。Necitumumab也是一种重组人抗EGFR单克隆抗体。在SQUIRE研究中，采用necitumumab联合化疗（吉西他滨和顺铂）对比单纯化疗一线治疗局部进展期或转移性鳞型NSCLC获得了1.6个月的OS改善和0.2个月的PFS改善。据此FDA批准了necitumumab与吉西他滨和顺铂联合方案用于一线治疗局部进展期或转移性鳞型NSCLC^[30]^。

### 3.2 维持/巩固治疗

在一线化疗4周期-6周期后，如肿瘤达到缓解或稳定，已有多项研究证实可予以继续或换药维持治疗（表 2），延长患者PFS和OS^[31-33]^。2013年Cai等^[34]^对同药或换药维持治疗的研究进行了荟萃分析。首先，按同药或换药维持划分，发现原药或换药维持均显著改善患者PFS，原药维持具有改善OS趋势，但无统计学意义，而换药维持能够显著改善患者OS。其次，按组织类型划分，非鳞癌亚组在OS和PFS方面均有显著改善，但鳞癌亚组并没有看到OS和PFS显著获益。Kulkarni等^[35]^对NSCLC维持治疗获益人群进行回顾和荟萃分析，研究共纳入14项维持治疗的随机对照试验。得出最佳OS获益人群为接受培美曲塞维持治疗的非鳞NSCLC患者（HR: 0.74; 95%CI: 0.64-0.86; *P* < 0.000, 1），鳞癌患者也无OS显著获益，这一结果与已有研究^[32]^的荟萃结果相一致。对于不可手术局部晚期NSCLC患者，同步放化疗是目前的标准治疗，尽管采用了先进的现代三维适形和调强放射治疗技术降低了心肺毒性，但OS也仅为17个月-27个月^[36-38]^。PACIFIC研究评估了局部晚期不可切除的NSCLC患者接受了标准的同步放化疗后，观察durvalumab（一种阻断PD-1/PD-L1结合的IgG1型单克隆抗体）维持治疗的疗效和安全性^[39]^。该试验共入组713例患者，在完成同步放化疗且达到SD以上疗效之后，以2:1的比例随机接受Durvalumab维持治疗或安慰剂治疗，持续12个月。结果表明Durvalumab组的中位PFS为16.8个月，远高于安慰剂组的5.6个月，HR为0.52。亚组分析显示EGFR野生型或状态未知人群较*EGFR*突变人群，PFS获益更有优势（亚组HR：0.47 *vs*全组HR：0.76），OS数据尚未成熟。因此，对于局部晚期不可切除的NSCLC患者放化疗后接受PD-1/PD-L1抗体免疫治疗可能成为一种新的选择，尤其是EGFR野生型患者。当然目前尚缺乏头对头比较免疫检查点抑制剂维持或巩固治疗EGFR野生型和*EGFR*突变型NSCLC的生存数据。

### 3.3 二线/后线治疗

TAX317/320研究奠定了多西他赛在NSCLC二线治疗的标准地位^[40, 41]^，而JMEI研究也奠定了培美曲赛在非鳞型NSCLC二线治疗中的地位^[42]^。在*EGFR*基因状态未选择的患者中，INTEREST和TITAN研究显示，EGFR-TKI（厄洛替尼、吉非替尼）与传统的标准二线化疗（多西他赛、培美曲赛）疗效相似。回顾性分析表明，厄洛替尼二线治疗EGFR野生型NSCLC也有一定疗效^[43-45]^。对于体力状态差、无法耐受化疗的患者，厄洛替尼成为这部分NSCLC二线治疗的选择之一。然而，后续多项研究（DELTA, TAILOR, CTONG0806）显示，对于*EGFR*野生型NSCLC患者，二线化疗（多西他赛、培美曲赛）无论是ORR和PFS都显著优于EGFR-TKI治疗^[46, 47]^。在Ⅲ期LUX-Lung 8试验研究中，直接比较阿法替尼和厄洛替尼在一线化疗后发生进展的晚期肺鳞癌患者中接受这两种EGFR靶向药物的疗效和安全性，结果显示，阿法替尼治疗可使死亡风险明显降低（19%），并使患者的中位生存期延长达7.9个月，超过厄洛替尼（6.8个月）；因此对于美国东部肿瘤协作组（Eastern Cooperative Oncology Group, ECOG）评分2分的晚期肺鳞癌患者，阿法替尼成为二线治疗的推荐选择之一^[48]^。

免疫治疗已经成为一线化疗失败后新的标准二线治疗推荐。目前FDA已经批准上市的免疫检查点类药物，如纳武单抗（nivolumab）、帕姆单抗（pembrolizumab）（限于PD-L1≥1%患者）和阿特珠单抗（atezolizumab），无论是鳞癌还是腺癌二线治疗OS都显著优于传统化疗药物多西他赛，中位OS延长3个月-4个月^[49-52]^。一项网络荟萃分析研究对EGFR野生型或状态未知的进展期NSCLC的不同二线治疗方法进行了荟萃分析，共纳入102项随机对照临床试验的36, 058例患者，结果发现，纳武单抗、帕姆单抗、阿特珠单抗及厄洛替尼联合培美曲赛能够更有效的延长患者生存^[53]^。仍有许多免疫治疗联合化疗的临床研究正在进行。

雷莫芦单抗是一种人血管内皮生长因子受体2（VEGFR2）拮抗剂。一项Ⅲ期随机对照临床试验（REVEL研究）对比了雷莫芦单抗联合多西他赛与多西他赛单药二线治疗转移性NSCLC患者的疗效，结果发现联合组中位PFS延长1.5个月，中位OS延长1.4个月^[54]^。

## 4 Pan-negative型NSCLC的展望

随着分子检测技术的发展，一些原本定义为pan-negative型NSCLC的患者也检测到某种驱动基因变异，这种患者可以给予相应的靶向药物治疗。Ali等^[58]^通过NGS法检测了386例肺癌FFPE（石蜡包埋组织）样本，发现在248例（68%）样本中至少1个基因变异会被目前的“热点分析”所遗漏。Drilon等^[59]^对35例pan-negative型（*EGFR*、*KRAS*、*NRAS*、*BRAF*、*HER2*、*PIK3CA*、*MEK1*阴性及*ALK*融合均阴性）不吸烟肺腺癌进行了RT-PCR或NGS检测，31%患者存在*RET*或*ROS1*融合，其中15%（5/34）患者存在ROS1融合，1例患者应用克唑替尼（crizotinib）达到PR；15%（5/33）存在RET融合，其中3例患者接受了卡博替尼（cabozantinib）治疗，2例达到了PR。除了检测技术的原因之外，pan-negative状态还应考虑到空间和时间异质性的影响。

总之，尽管目前化疗仍是pan-negative型NSCLC治疗的基石，但抗PD-1/PD-L1单抗等免疫检查点类药物已成为一线化疗失败后的二线治疗新的标准，甚至在PD-L1高表达肺癌患者的一线治疗中显示出了显著的OS获益以及良好的安全性。可以预见，将来必定会有更多的驱动基因及肿瘤相关的异常信号通路被发现，开发出相应特异性的靶向药物和信号通路抑制剂。今后研究的方向将是如何更好的发挥免疫检查点药物的优势，努力寻找疗效预测分子标志物以提高免疫治疗的精准性。此外，要积极探索免疫检查点药物与其他治疗方式或其他药物的联合应用，包括免疫药物之间的联合，免疫药物与放疗的联合，免疫与化疗和抗血管药物之间的联合，以及免疫治疗抑制剂（CTLA-4抑制剂和杀伤细胞免疫球蛋白样受体KIR抑制剂）和免疫治疗的联合，以期进一步提高pan-negative型NSCLC治疗疗效，使之成为新的标准治疗。
